# Chicago Medical Society

**Published:** 1869-03

**Authors:** 


### CHICAGO MEDICAL SOCIETY.

FRIDAY EVENING, Jan. 15, 1869.

The Society was called to order, President Marguerat in the chair.

Secretary Macdonald then read the proceedings of the last meeting, which were duly approved, and ordered to be placed on file.

The Society next proceeded to the discussion of "the therapeutical action and value of mercury and its preparations."

Dr. Loverin opened the discussion, by reading an interesting history of the mineral, where obtained; its different preparations, and modes of preparing them. Entered into the therapeutical uses of the most common preparations, including hydrargyri chloridum mite; hydrargyri bichloridum; hydrargyri ammoniatum; hydrargyri iodidum; hydrargyri biniodidum; unguentum hydrargyri nitrico-oxidi; together with the strong and mild preparations of unguentum hydrargyri.

Dr. Loverin believes mercury very useful in æsthenic inflammations.

Dr. Bevan says that for the past four or five years he has been in the habit of treating acne with an ointment containing of hydr. biniodidi g. iij. to vij. to ℥j. of lard. Says that 5 grs. to the ounce will sometimes produce vesication. In some cases, where treatment was continued from two to eight months, has seen chronic acne of two years' standing entirely cured. In addition, it is sometimes necessary to give cod-liver oil and tonics. Has seen cases recover under this treatment when there was no benefit derived from sulphur, camphor, and rose-water, as recommended by Wilson.

Dr. Wanzer said he had treated a chronic ulcer of the leg by administering ℞. Hydr. bichlor. gr. $\frac{1}{8}$, Opii gr. $\frac{3}{4}$, three times a day, with perfect success, and that he is curing one at present. Says that he recommended the above treatment to the physician in charge of the Poor-House, who also treated several ulcers of similar character with success, and has never seen but one exception. Thinks the way calomel cures inflammation is by diminishing the plasticity of the blood.

Dr. Quails asked Dr. Wanzer if he made any distinction between the specific and non-specific form of ulcer in his treatment?

Dr. W. replied that he did not.

Dr. Weller was permitted to make some remarks. Stated that he had treated acne successfully with the bychloride hydrargyri; also used it in diphtheritic ophthalmia and diphtheria of the throat. Has used it in the abortive treatment of felons, by preventing suppuration. Uses a solution consisting of bichlor. hydr. gr. j. to alcohol dilute ℥j. Cited a case which had been progressing four days, in which, by the application of the solution on a piece of cloth, all swelling subsided in fourteen hours.

Dr. Davis says it has generally been stated that mercury diminishes the plasticity of the blood. Thinks it true of some of its preparations. But much depends on the way it is used. Does not think its action always the same. Thinks the use of bichlor. hydrargyri beneficial in chronic albuminuria or Bright's disease of the kidneys, when the blood is spanæmic. Speaks of its use by Dr. Johnson in the New York hospitals twenty years ago, in the latter-named disease; also of his own use, in combination with the tincture of cinchona, bichlor. hydr. gr. j. and tinct. cinchona ℥ij., of which a teaspoonful was given three times a day. After continuing several days, the tinct. of cinchona was increased to ℥iij., the dose being the same, with an intermission of three or four days, to prevent salivation. After two months, there was but a scanty precipitate of albumen to be found in the urine. In one year the patient had gained very much, and was able to work two-thirds of a day in a harvest-field, although not entirely free from the disease. Has employed it in a number of cases since, and in most cases with some benefit. Speaks of its beneficial use in irritable corneitic inflammation, charaterized by the red zone around the cornea, with little ulcers upon its surface, and great sensitiveness to light. The class of children affected by this disease are generally of the scrofulous diathesis. For twenty years has hardly met with a case but what has yielded under the following treatment:—

℞. Tinct. Cinchona,-------------------- ℥ij.
Bichlor. Hydr.,---------------------- gr. j.
*M.*

Twenty drops of which may be given to a child five or six years old, three times a day. They do not generally begin to derive benefit from the medicine until it has been given four or five days. The photophobia and irritability being removed in three or four weeks.

Sometimes it is necessary to use anodynes locally. For this he uses morphia, 4 grs. in water ℥j., dropped into the eye three times a day; also veratria, grs. iij. to ℥iv. dilute alcohol, which may be applied as a wash over the eyebrows each night and morning. After the inflammatory symptoms have disappeared, he often substitutes syrup of iodide of iron or of lime for the bichloride and tincture of cinchona.

After some brief remarks by Drs. Wanzer and Loverin, the discussion was closed, and the Society proceeded to Miscellaneous Business.

The President announced that at the last meeting there was an assessment of $1.00 made on each member of the Society.

Drs. Davis, Paoli, and Holmes were appointed on the "selection of proper subjects for discussion by the Society."

Dr. Davis wished to know if any members had information regarding the Bill now pending before the State Legislature to regulate medical practice.

Dr. Seely remarked, he had read of such in the city papers, and believed such a Bill should be passed. Upon his motion, Dr. Davis was appoidted a Committee to report on the subject at next meeting of the Society.

Society adjourned.

---

FRIDAY EVENING, Jan. 22, 1869.

The Society was called to order, President Marguerat in the chair.

Secretary Macdonald read the minutes of the last meeting of the Society, which were duly approved.

Dr. Clarke called for the law governing the non-attendance of members, which was read by the Secretary, setting forth, "that if a member was absent three successive meetings without notifying the Society of the necessity of his absence, he is liable to have his name stricken from the register as a member of the Society."

Dr. Reid reported several cases of neuralgia, which he had found quite obstinate in treatment:—

CASE I. A lady, who suffered great pain in her head towards morning, and truly paroxysmal in character. Gave quinine in large doses, and partially succeeded in allaying her sufferings. Then gave arsenious acid and Indian hemp, with marked improvement. She has been under treatment about three weeks, and is now in the use of iron, and nearly recovered.

CASE II. Old gentleman. Pain along the sciatic nerve. Bowels constipated. Gave quinia, which diminished pain somewhat. Tried arsenious acid, without much benefit. Upon purging, there were hard, impacted fæces passed, and the patient was better afterwards. Tried the Voltaic battery, with some benefit. Pain left ankle, and went to hip. Blisters were applied, and patient seemed to improve for a few days, but is now nearly as bad as ever.

Treated several cases successfully by the use of arsenic and quinine.

CASE III. Lady. Neuralgia of bladder every night. Gave purgative, when a great quantity of hardened fæces were passed. Gave quinia, with benefit. Gave Dr. Gross's colchicum and morphia treatment, which was followed by great disturbance of the bowels and severe purging. Patient improved rapidly. Thinks there is great benefit derived from purging.

Dr. Paoli says that he has used in these obstinate cases of neuralgia the permanganate of potassa, in $\frac{1}{4}$-gr. doses, three times a day, with marked benefit; also using a strong solution externaly.

Dr. Clarke says that he has had a number of cases of rheumatism of late, also a case of sciatica, in which quinia and arsenic had been administered, without benefit. Most of the cases were very obstinate, but had very good results from citrate of potassa.

Dr. Hamill reported the case of a lady who had neuralgia of crural nerve, which was paroxysmal in character. An hour before the paroxysm was expected, he introduced, by hypodermic injection, morphia, gr. $\frac{1}{4}$, into the inner part of the thigh. The paroxysm was arrested, but it left the pain diffused over a larger surface. Then gave

℞. Bromide Potassa,------------------ ʒss.

Tinct. Gelseminum,---------------- gtt. v., every three hours, when the pain was overcome. Treated ordinary cases of sciatica by the use of tinct. guaiac and phytolacca, equal parts; a teaspoonful of which may be given every four or six hours.

Dr. Loverin said he had treated a case of sciatica successfully by the use of cathartics, anodynes, Fowler's solution, and quinine. Says he has heard recommended large doses of the carbonate of iron, and also nydroganic acid.

Dr. Davis made the following report regarding the Medical Bill now before the Legislature. Stated that he had written a letter to Springfield requesting a copy of the bill, but he was unable to obtain it. He believed, from the knowledge he had received from his friend in Springfield, that the principal bill now before the Legislature is the one introduced by Dr. Edgar, the substance of which is: that it requires every man proposing to practice medicine in the State of Illinois to show that he has received a medical education; and that such persons having no diploma are to be examined by a medical board, and, if found competent, will receive a license, on payment of $25; and those physicians having diplomas, and presenting the same, upon payment of $5, would be registered. He was of the opinion that the two bills in the respective houses were somewhat the same; the objəct being to restrict the practice of medicine to such persons who had actually received a competent medical education. By this means, the community would be, in a great measure, protected against imposition. He also considered that so far as protecting the medical faculty was concerned, it only imposed upon them an additional burden, in the interest of the public. He hoped that the Society would take action in favor of having the bill passed and made a law, as the profession at Jacksonville and Springfield are evidently laboring to secure its passage.

Dr. Wickersham stated that he was opposed to the bill, and announced himself a radical on the question. He had not given twenty years of his life for the privilege of associating himself with *quackery*. He said he was surprised, and regretted, to see Dr. Davis favor the bill. He believed if our Legislature would pass a law excluding from the papers and periodicals quack medical advertisements, it would confer a great favor upon the public; but was entirely opposed to the Societie's taking any action on this matter whatever; for he was firm in the belief that nearly all medical advertisers had diplomas from medical schools, and that this very bill would protect them in their impositions, for they could easily obtain certificates from the State Board.

Dr. Clarke expressed his views in favor of the bill, and believes we are in much need of such a law for protection of the people. Does not think more than one-tenth of the advertising quacks are graduates of our regular medical colleges.

Dr. Reid hoped the Society would act only upon a more thorough knowledge of the details of the bill.

Dr. Davis remarked that he was not a strenuous advocate of legislation for the profession, and said that any fair rendering of the bill would cut of those who were not legitimate members of the profession. The bill simply refers to medical education and medical practice, without recognizing homeopathy, or any other *ism*.

Dr. Hamill did not favor the further action in the matter, until the Society knew more respecting it; and moved that the matter be laid on the table.

Dr. Fitch hoped the Society would take immediate action, and adopt measures to secure the passage of the bill.

Dr. Wicksrsham was in favor of the Societie's passing a resolution against the enactment of such a law as the one proposed. He believed this was the age of medical, political, and religious quackery, and, as such, should be recognized and acted upon.

Dr. Paoli believed the profession should be entirely independent of all isms. He hoped all future action of the Society would be based on a copy of the bill.

Dr. Davis said he was confident that no legislature would pass a law creating a board of medical examiners, to examine those recommended by the medical profession.

The motion of Dr. Hamill being called for, the whole matter was laid on the table.

Society adjourned.

